# Medical Congress of Italy

**Published:** 1848-07

**Authors:** 


*Medical Congress of Italy.*—Amongst the papers read at the scientific meeting, held at Venice, in September, 1847, (Section of Medicine and Surgery,) was a communication by Dr. Verga Andrea, of Milan, on steracutine. Such is the name given, some time ago, by Professor Semmola, of Naples, to a fatty substance of a greenish colour, which children at the breast sometimes evacuate, whilst under the influence of some nervous attack, as colic, eclampsia, epilepsy, syncope, &c.; and which the Neapolitan professor attributed to the imperfect digestion of the butyrous portion of the milk. Dr. Verga's researches tend to elucidate the following points:—

1. That children at the breast void such solid fatty concretions, when under the influence of certain diseases, is correct. These concretions are more or less rounded, transparent in their periphery, soft to the touch, insoluble in water, but soluble in alcohol.

2. This substance is not voided by sucking children only; they have been seen at six and seven years old, and even in adults.

3. Nervous diseases are not the only affections connected with it; the author has observed it in measles and meningitis.

4. This substance is almost exclusively composed of stearine, and cholesterine exists in it in a very small proportion.

5. There is no constant and regular connexion between these evacuations and the progress of the diseases during which they may be observed.

6. Like other excretions, this may prove critical and salutary, or it may be merely symptomatic, and of a bad omen.

A paper, read by Dr. Calmarino, of Naples, contained the following quaint proposal for the establishment in town, as well as in the country, of a Female Hygienic Society, under the immediate direction of the medical men of the place, in order—

1. To lead the young brides to the nuptial bed.

2. To watch over their first pregnancy, directing the young wives what they ought to do or to avoid.

3. To be present at the first delivery; to watch over careless midwives, and give proper advice.

4. To induce the mothers to nurse their children themselves.

5. To teach them the proper way to get their offspring safely over the period between birth and dentition.

6. To give good counsel to young girls as to the developement of puberty, and concerning the care they should take of themselves.

7. To instruct all young girls on the eve of marriage.

(The secretary of the Section does not say what the meeting thought of this curious proposal.)—*London Lancet.*

